# Development of a Multiplex Loop-Mediated Isothermal Amplification Assay for Diagnosis of *Plasmodium* spp., *Plasmodium falciparum* and *Plasmodium vivax*

**DOI:** 10.3390/diagnostics11111950

**Published:** 2021-10-20

**Authors:** Woong Sik Jang, Da Hye Lim, YoungLan Choe, Hyunseul Jee, Kyung Chul Moon, Chaewon Kim, Minkyeong Choi, In Su Park, Chae Seung Lim

**Affiliations:** 1 Emergency Medicine, College of Medicine, Korea University Guro Hospital, Seoul 08308, Korea; vector18@korea.ac.kr (W.S.J.); cmooner@korea.ac.kr (K.C.M.); 2 Departments of Laboratory Medicine, College of Medicine, Korea University Guro Hospital, Seoul 08308, Korea; ldh9692@korea.ac.kr (D.H.L.); ylan821@naver.com (Y.C.); jhs603@korea.ac.kr (H.J.); chaewon.kim1111@gmail.com (C.K.); min508k@naver.com (M.C.); in2269@korea.ac.kr (I.S.P.)

**Keywords:** *Plasmodium* spp, *Plasmodium falciparum*, *Plasmodium vivax*, LAMP, multiplex LAMP

## Abstract

Malaria, caused by the parasite *Plasmodium* and transmitted by mosquitoes, is an epidemic that mainly occurs in tropical and subtropical regions. As treatments differ across species of malarial parasites, there is a need to develop rapid diagnostic methods to differentiate malarial species. Herein, we developed a multiplex malaria Pan/Pf/Pv/actin beta loop-mediated isothermal amplification (LAMP) to diagnose *Plasmodium* spp., *P. falciparum*, and *P. vivax*, as well as the internal control (IC), within 40 min. The detection limits of the multiplex malaria Pan/Pf/Pv/IC LAMP were 1 × 10^2^, 1 × 10^2^, 1 × 10^2^, and 1 × 10^3^ copies/µL for four vectors, including the 18S rRNA gene (*Plasmodium* spp.), lactate dehydrogenase gene (*P. falciparum*), 16S rRNA gene (*P. vivax*), and human actin beta gene (IC), respectively. The performance of the LAMP assay was compared and evaluated by evaluating 208 clinical samples (118 positive and 90 negative samples) with the commercial RealStar^®^ Malaria S&T PCR Kit 1.0. The developed multiplex malaria Pan/Pf/Pv/IC LAMP assay showed comparable sensitivity (100%) and specificity (100%) with the commercial RealStar^®^ Malaria S&T PCR Kit 1.0 (100%). These results suggest that the multiplex malaria Pan/Pf/Pv/IC LAMP could be used as a point-of-care molecular diagnostic test for malaria.

## 1. Introduction

Malaria, which is caused by the *Plasmodium* protozoan and transmitted by mosquitoes, is an epidemic that predominantly occurs in tropical and subtropical regions [1]. Malaria infection has mainly been reported in Africa and several other countries, and more than 40% of the world’s population remains at risk from various malarial species [2]. According to the World Health Organization (WHO), 409,000 malaria deaths were reported worldwide in 2019 [3]. *Plasmodium* species that infect humans include *Plasmodium falciparum*, *Plasmodium vivax*, *Plasmodium malariae*, *Plasmodium ovale*, and *Plasmodium knowlesi* [4]. *P. falciparum* accounts for 99.7% of malaria cases in Africa and causes fatal symptoms. *P. vivax* induces weaker symptoms than *P. falciparum* but is known to occur worldwide and has a 75% infection rate in the Americas [3]. Malaria infections commonly cause frequent fevers, anemia, and an enlarged spleen; however, treatments differ for each species [5]. Malaria treatments are largely divided into chloroquine and artemisinin antibiotics [6]. *P. vivax* and *P. ovale* are treated using chloroquine antibiotics; *P. falciparum*, *P. malariae*, and *P. knowlesi* are treated with artemisinin antibiotics [7]. These five types of human-affecting Plasmodia are responsible for an infectious disease that can be cured if appropriate treatments are employed. However, if *P. falciparum* is not detected early, it can lead to severe disease and death [8]. Therefore, it is crucial to diagnose malaria promptly during early stages and distinguish among malaria species [9].

For malaria detection, currently available diagnostic methods include blood smears, the application of a rapid diagnostic kit (RDT), and molecular diagnostics. Blood smear, a representative diagnosis sample for malaria, is simultaneously performed with thick- and thin-layer smears, followed by Giemsa staining, in order to identify the presence of *Plasmodium* species under a microscope [10]. However, blood smears are time-consuming and require trained specialists because the staining process takes 45 min [11] and a trained microscopist consumes 15–30 min for microscopic examination of a single blood slide [11]. Furthermore, with thick-layer smears, it is difficult to identify the type of malaria, and with thin-layer smears, it can be challenging to confirm the diagnosis if the infection density is low [12]. RDT results can be confirmed within 20 min, but the sensitivity is lower than that of other diagnostic methods [13]. Polymerase chain reaction (PCR), a molecular diagnostic method, has the highest sensitivity and specificity among malaria diagnosis methods and can identify *Plasmodium*, as well as distinguish between species [14]. However, PCR takes approximately 3 h and requires expensive equipment with thermal cycling capabilities and skilled professionals [15]. Thus, several isothermal amplification methods for malaria diagnoses have been proposed as molecular diagnostic methods to overcome these limitations, including loop-mediated isothermal amplification (LAMP) [16], lateral flow recombinase polymerase amplification (LF-RPA) [17], and quantitative nucleic acid sequence-based amplification (QT-NASBA) [18]. Among these isothermal amplification methods, LAMP is the most extensively investigated method for malaria diagnosis [19,20,21,22]. LAMP requires four primers, containing six parts of the target gene sequence and two loop primers, that enhance the performance of the loop structure. This primer set reacts with the target gene to form a loop structure, thus robustly amplifying the target gene at 58–65 °C using Bst polymerase, a displacement DNA polymerase.

To date, a variety of LAMP detection methods have been developed, including colorimetric and fluorescence detection, detection using fluorescent probes, and detection using lateral flow devices [23]. Among these, detection methods using fluorescent probes can be adapted to multiplex LAMP assays. Multiplex LAMP methods that use currently available probes include the methylation-specific LAMP (MS-LAMP), assimilating probe-LAMP, fluorescence of loop primer upon self-enriching-LAMP (FLOS-LAMP), detection of amplification by release of quenching (DARQ), quenching of unincorporated amplification signal reporters (QUASR), and molecular beacon LAMP (MB-LAMP) [24,25,26,27,28,29].

In the present study, we developed a multiplex malaria Pan/Pf/Pv/IC LAMP method to detect all *Plasmodium* species (Pan), *P. falciparum* (Pf), *P. vivax* (Pv), and the internal control (IC; human beta-actin), based on a slightly modified DARQ probe method. The limit of detection (LOD) test for the multiplex malaria Pan/Pf/Pv/IC LAMP assay was performed using four *Plasmodium* species, namely *P. falciparum*, *P. vivax*, *P. malariae*, and *P. ovale*. In addition, the performance of the multiplex Pan/Pf/Pv/IC LAMP assay was compared with that of the commercial RealStar^®^ Malaria S&T PCR Kit 1.0.

## 2. Materials and Methods

### 2.1. Clinical Samples and DNA Extraction

Clinical whole blood samples were collected from patients infected with malarial parasites at the Korea University Guro Hospital from June 2000 to July 2017. All clinical samples were diagnosed by microscopic examination of Giemsa-stained blood smears and in-house PCR tests at the Korea University Guro Hospital. In total, 208 whole blood specimens were used in the present study, including 118 positive specimens of four types of malaria (61 *P. falciparum*, 54 *P. vivax*, 2 *P. malariae*, and 1 *P. ovale*) and 90 negative specimens. DNA extraction from the 208 whole blood samples was performed using the NX-48 Viral NA Kit, with a Nextractor NX-48 system (Genolution, Seoul, Korea), according to the manufacturer’s instructions. DNA was stored at −50 °C. All tests were performed in a blinded manner, with the operator unaware of any previous test results. The study was conducted in accordance with the guidelines of the Declaration of Helsinki and was approved by the Institutional Review Board of Korea University Guro Hospital (2017GR0769 (8 March 2017), 2020GR0451 (7 October 2020)).

### 2.2. Primer Design

Before use in the LAMP assays, *P. falciparum* LAMP primer sets were designed for the lactate dehydrogenase (LDH) gene of *P. falciparum* and assessed for specificity via a BLAST search of sequences in GenBank (National Center for Biotechnology Information [NCBI], Bethesda, MD, USA). *P. falciparum* LAMP primers, including two outer primers (forward primer F3 and backward primer B3), two inner primers (forward inner primer FIP and backward inner primer BIP), and two loop primers (forward loop primer LF and backward loop primer LB), were designed using the Primer Explorer software (Version 4; Eiken Chemical Co., Tokyo, Japan). To detect all malaria species and *P. vivax*, we used malaria Pan and *P. vivax* LAMP primer sets, designed by Mohon et al. and Chen et al., respectively [30,31]. For the IC, we used the previously designed LAMP primer set for the actin beta gene [32]. A multiplex LAMP assay was designed using two types of additional oligonucleotide sequences at the 5′ LB primers for probing and complementary oligonucleotide sequences for quenching. For the multiplex LAMP assay, FAM, HEX, Texas Red, and Cy5 fluorescence tagging probes were used for Malaria Pan, *P. falciparum*, *P. vivax*, and IC, respectively. All LAMP primers and probes were synthesized by Macrogen Inc. (Seoul, Korea; Table 1).

### 2.3. Multiplex Malaria Pan/Pf/Pv/IC LAMP Assay

The multiplex malaria Pan/Pf/Pv/IC LAMP assay was performed using a Mmiso DNA amplification kit (M monitor, Daegu, South Korea). The LAMP reaction mixture comprised of 12.5 µL of 2x reaction buffer, 0.625 µL of malaria Pan LAMP primer mix, 0.625 µL of *P. falciparum* LAMP primer mix, 1.25 µL of *P. vivax* LAMP primer mix, 0.6 µL of IC LAMP primer mix, 720 nM quencher 1 solution, 216 nM quencher 2 solution, 2 µL of enzyme mix, and 5 µL of sample DNA (final reaction volume: 25 μL). The LAMP primer mix was composed of 4 µM of two outer primers (F3 and B3), 32 µM of two inner primers (FIP and BIP), 10 µM of loopF primer, 6 µM of loopB primer, and 4 µM of loopB probe. The LAMP assay was run on a CFX 96 Touch Real-Time PCR Detection System (Bio-Rad Laboratories, Hercules, CA, USA) at 60 °C for 40 min. In addition, a negative control (human serum RNA and distilled water) was used as the baseline. The positive signal was determined by examining whether the signal was steep or gradual, considering the baseline, as the baseline of the LAMP assay was unstable when compared with quantitative PCR (qPCR) or RT-PCR.

### 2.4. Real-Time Reverse Transcription (RT)-PCR

The RealStar^®^ Malaria Screen & Type PCR Kit 1.0 (Altona Diagnostics, Hamburg, Germany) was performed with the CFX96 Touch Real-Time PCR Detection System (Bio-Rad Laboratories, Hercules, CA, USA), according to the manufacturer’s protocol. Thermocycling parameters specified in the kit were as follows: inactivation at 95 °C for 2 min, 45 cycles of denaturation at 95 °C for 15 s, annealing with fluorescence detection at 58 °C for 45 s, and extension at 72 °C for 15 s. The RealStar^®^ Malaria Screen & Type PCR Kit 1.0 was used with two tubes, one tube to diagnose *P. falciparum* and *P. vivax*, as well as the other tube to diagnose *P. knowlesi*, *P. malariae*, and *P. ovale*.

### 2.5. Limit of Detection (LOD) Tests of the Multiplex Malaria Pan/Pf/Pv/IC LAMP Assay

The LOD of the multiplex malaria Pan/Pf/Pv/IC LAMP was determined using pTOP Blunt V2 vectors, including the 18S rRNA partial gene sequences of *Plasmodium* spp., LDH partial gene sequences of *P. falciparum*, the 16S rRNA gene partial sequences of *P. vivax*, and beta-actin partial gene sequence of humans. All vectors were constructed by Macrogen, Inc. The vectors were serially diluted 10-fold, from 1.0 × 10^7^ copies/μL to 1.0 × 10^1^ copies/μL, to determine the LOD of the multiplex malaria Pan/Pf/Pv/IC LAMP assay. In addition, the LOD of malaria Pan/Pf/Pv/IC LAMP was tested with serially diluted clinical samples (10^−1^ to 10^−6^) infected with *P. falciparum*, *P. vivax*, *P. malariae*, and *P. ovale*. The LOD of the multiplex malaria Pan/Pf/Pv/IC LAMP for clinical malaria samples was compared with that of the RealStar^®^ Malaria Screen & Type PCR Kit 1.0.

## 3. Results

### 3.1. Optimization of the Multiplex Malaria Pan/Pf/Pv/IC LAMP Primer Set

**Figure 1:**
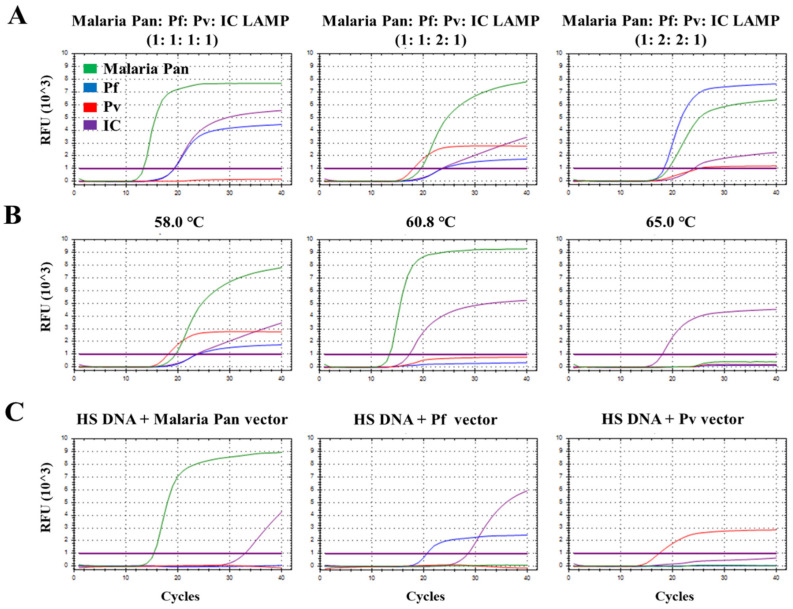
Optimization of the multiplex malaria Pan/Pf/Pv/IC LAMP primer set. (**A**) Different concentration ratios of the malaria Pan, *Plasmodium falciparum*, *Plasmodium vivax*, IC primer sets (1:1:1:1, 1:1:2:1, and 1:2:2:1, respectively) for malaria Pan, *P. falciparum*, *P. vivax*, and IC vectors. (**B**) Temperature gradient tests (58/60.8/65 °C) of the multiplex malaria Pan/Pf/Pv/IC LAMP assay. (**C**) Performance of malaria Pan/Pf/Pv/IC LAMP assay against HS DNA spiked with malaria pan vector, HS DNA spiked with *P. falciparum* vector, and HS DNA spiked with *P. vivax vector*. IC, internal control; LAMP, loop-mediated isothermal amplification. The color of the curves is the same in all panels and the meaning of curve colors is indicated in Panel A.

To optimize the multiplex malaria Pan/Pf/Pv/IC LAMP primer set, different concentration ratios of the malaria Pan, Pf, Pv, and IC primer sets (1:1:1:1, 1:1:2:1, and 1:2:2:1, respectively) were evaluated against the synthetic malaria Pan, *P. falciparum*, *P. vivax*, and human actin beta vectors (10^7^ copies; ratio of 1:1:1:1) (Figure 1A). Three signals (malaria Pan, Pf, and IC) were detected at a ratio of 1:1:1:1, whereas four signals (malaria Pan, Pf, Pv, and IC) were detected at ratios of 1:1:2:1 and 1:2:2:1. Among the three ratios of the LAMP primer set, the ratio of 1:1:2:1 for the multiplex malaria Pan/Pf/Pv/IC LAMP primer set exhibited the lowest Ct values (19.55, 23.83, 18.12, and 18.12) for malaria Pan, *P. falciparum*, *P. vivax*, and IC vectors, respectively. Thus, the ratios of malaria Pan, Pf, Pv, and IC LAMP primer set (1:1:2:1) were optimum for the multiplex malaria Pan/Pf/Pv/IC LAMP assay. Next, temperature gradient tests (58/60.8/65 °C) were performed to determine the optimal temperature for the multiplex malaria Pan/Pf/Pv/IC LAMP assay (Figure 1B). Among the three different temperatures, the LAMP assay performed at 58 °C showed the lowest Ct values and relative fluorescence unit (RFU) of all fluorescence channels (Ct/RFU, malaria Pan: 19.55/8132, Pf: 23.83/1810, Pv: 18.12/2689, and IC: 23.57/4367). Figure 1C presents the multiplex malaria Pan/Pf/Pv/IC LAMP assay, performed using human serum DNA samples spiked with malaria Pan, *P. falciparum*, and *P. vivax* vectors (10^7^ copies) under optimum conditions. The ratio of the malaria Pan, Pf, Pv, and IC LAMP primer sets was 1:1:2:1, and the LAMP assay was performed at 58 °C.

### 3.2. Limit of Detection (LOD) of the Multiplex Malaria Pan/Pf/Pv/IC LAMP Assay

**Figure 2:**
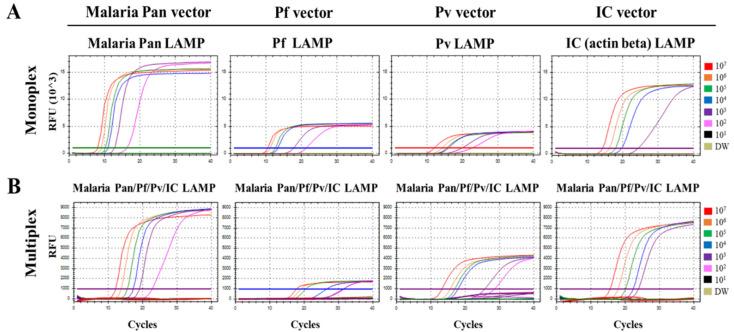
Limit of detection (LOD) test for the monoplex and multiplex malaria Pan/Pf/Pv/IC LAMP primer set. (**A**) The monoplex malaria Pan, *Plasmodium falciparum*, *Plasmodium vivax*, and IC LAMP primer sets (left, middle, and right panels, respectively). (**B**) Multiplex malaria Pan/Pf/Pv/IC LAMP primer set, respectively. The monoplex and multiplex malaria Pan/Pf/Pv/IC LAMP assays were performed with synthetic malaria Pan, *P. falciparum*, *P. vivax*, or beta-actin vectors, ranging from 10^7^ to 10^1^ RNA copies/μL. Colors (red, orange, green, blue, purple, pink, black, and olive) indicate vector copy numbers/μL (1.0 × 10^7^ to 1.0 × 10^1^ copies/μL) and distilled water (DW; negative control). The LOD test was performed in duplicate. IC, internal control; LAMP, loop-mediated isothermal amplification.

The analytical sensitivity of the multiplex malaria Pan/Pf/Pv/IC LAMP assay was compared with that of the corresponding monoplex LAMP primer sets against synthetic pan, Pf, Pv, and IC vectors, ranging from 10^7^ to 10^1^ DNA copies/μL (Figure 2, Table 2). The monoplex malaria Pan, Pf, Pv, and IC LAMP revealed an LOD of 1 × 10^2^, 1 × 10^2^, 1 × 10^2^, and 1 × 10^3^ copies/µL, respectively. In the multiplex malaria Pan/Pf/Pv/IC LAMP assay, the LOD results for the four channels were the same as those for the corresponding monoplex LAMP assays; the Ct values of the multiplex malaria Pan/Pf/Pv/IC LAMP assay were delayed, when compared with those of the corresponding monoplex LAMP assay. Furthermore, the LOD of the LAMP assay was compared with that of monoplex LAMP and commercial RealStar^®^ Malaria Screen & Type PCR Kit 1.0 (Altona Diagnostics, Hamburg, Germany) using serially diluted *P. falciparum*, *P. vivax*, *P. malariae*, and *P. ovale* clinical samples (clinical whole blood DNA sample dilution range of 10^−1^ to 10^−6^; Table 3). For the *P. falciparum* clinical samples, monoplex malaria Pan and *P. falciparum* primer sets showed detection limits of 10^−4^ and 10^−3^, respectively. The multiplex malaria Pan/Pf/Pv/IC LAMP assay showed an LOD of 10^−4^ (pan), 10^−4^ (Pf), and 10^−2^ (IC), whereas RealStar^®^ Malaria Screen & Type PCR Kit 1.0 showed an LOD of 10^−5^. For the *P. vivax* clinical samples, the monoplex malaria Pan and Pv LAMP primer sets showed LODs of 10^−4^ and 10^−2^, respectively. The multiplex malaria Pan/Pf/Pv/IC LAMP assay presented LODs of 10^−3^ (pan), 10^−3^ (Pv), and 10^−2^ (IC), and the RealStar^®^ Malaria Screen & Type PCR Kit 1.0 showed the same detection limits (10^−3^). For the *P. malariae* and *P. ovale* clinical samples, the monoplex malaria Pan primer set showed LODs of 10^−4^ and 10^−1^, respectively. The multiplex malaria Pan/Pf/Pv/IC LAMP assay showed detection limits of 10^−3^ (pan) and 10^−1^ (pan) for *P. malariae* and *P. ovale*. The RealStar^®^ Malaria Screen and Type PCR Kit 1.0 showed detection limits of 10^−5^ and 10^−1^ for *P. malariae* and *P. ovale* clinical samples, respectively.

### 3.3. Comparison of Clinical Performance between the Multiplex Malaria Pan/Pf/Pv/IC LAMP and RealStar^®^ Malaria Screen & Type PCR Kit 1.0 Using Clinical Samples

To confirm the clinical performance of the multiplex malaria Pan/Pf/Pv/IC LAMP assay, the assay sensitivity and specificity were compared with those of RealStar^®^ Malaria Screen & Type PCR Kit 1.0 for *P. falciparum* (n = 61), *P. vivax* (n = 54), *P. malariae* (n = 2), *P. ovale* (n = 1), and healthy patient (n = 90) whole blood specimens (Table 4). For *P. falciparum* clinical samples (n = 61), the sensitivities for *P. falciparum* and IC were 100% and 95.08%, respectively, for the RealStar^®^ Malaria Screen and Type PCR Kit (1.0). The sensitivity of the multiplex malaria Pan/Pf/Pv/IC LAMP assay was 100% in the malaria Pan channel (FAM), 100% in the Pf channel (HEX), and 75.41% in the IC channel (Cy5). The cross-reactivity of the Pv channel of the multiplex malaria Pan/Pf/Pv/IC LAMP assay was 100%, whereas that of the Pv channel of the RealStar^®^ Malaria Screen & Type PCR Kit 1.0 was 98.36% against *P. falciparum* clinical samples (n = 61). For *P. vivax* clinical samples (n = 54), the sensitivities for *P. vivax* and IC were 100% with the RealStar^®^ Malaria Screen and Type PCR Kit 1.0; the sensitivities of the multiplex malaria Pan/Pf/Pv/IC LAMP assay were 100% in the malaria Pan channel (FAM), 100% in the Pv channel (HEX), and 81.48% in the IC channel (Cy5). The Pf channels of the two assays showed no cross-reactivity with the *P. vivax* clinical samples. For the *P. malariae* (n = 2) and *P. ovale* clinical samples (n = 1), the sensitivities and specificities of both assays were 100%.

## 4. Discussion

The accurate classification of malaria species during the early stage of diagnosis is valuable for the proper treatment of infected patients and prevention of overexposure to antimalarial drugs, as treatment policies for different species tend to vary, such as artemisinin-based combination therapy (ACT) for *P. falciparum* and chloroquine for non-falciparum species [7,33]. Currently, microscopy is the gold standard diagnostic method for malaria (including the classification of malaria species); however, an expert with a high level of training and experience is needed for accurate diagnosis, especially for species identification [34,35].

In the present study, we developed multiplex differential diagnostic tools to classify the malaria Pan, *P. falciparum*, and *P. vivax*. Developing multiplex LAMP assays lacking non-specific signals is substantially more difficult than developing multiplex qPCR, as one LAMP assay uses six or seven primer/probe mixtures, and combinations of 2–4 LAMP sets can potentially result in the non-specific amplification of several primers. In particular, in the case of LAMP with the DARQ probe method, the probe sequence corresponding to the binding region of the LAMP product needs to be designed with consideration of the low homology with sequences of other primers and LAMP products, given that it induces non-specific signals by probe-primer or probe-LAMP product interaction. Herein, the multiplex malaria Pan/Pf/Pv/IC LAMP assay was designed by combining a newly designed Pf LAMP primer set with Malaria pan and *P. vivax* LAMP primer sets, designed by Mohon et al. and Chen et al., respectively [30,31]. This combination of four LAMP primer sets (malaria Pan, Pf, Pv, and IC) exhibited no cross-reactivity with each other, as well as 100% specificity for non-infected samples and distilled water. In the LOD test for *P. falciparum* and *P. ovale*, the multiplex malaria Pan/Pf/Pv/IC LAMP showed a LOD 10-fold and 100-fold higher than that of the commercial RealStar^®^ Malaria S&T PCR Kit 1.0, respectively. For *P. vivax* and *P. malariae*, the LOD of the LAMP assay was the same as that obtained with the commercial kit. Notably, the multiplex malaria Pan/Pf/Pv/IC LAMP assay showed a slightly higher LOD than the commercial PCR kit. However, after assessing 208 clinical samples, the multiplex malaria Pan/Pf/Pv/IC LAMP assay revealed sensitivity (100%) and specificity (100%) similar to those detected with the commercial RealStar^®^ Malaria S&T PCR Kit 1.0 (100%).

As the isothermal amplification method does not require an expensive temperature control device to change the temperature for target gene amplification, the isothermal amplification method has the advantage of easy application for on-site diagnosis, which is difficult to implement in general PCR [15,36]. Unfortunately, the multiplex LAMP assay developed using fluorescence is limited, in terms of field applications [37]. Isothermal amplification, or a portable PCR device for detecting multiple fluorescence signals, is required to utilize the multiplex LAMP assay for point-of-care testing (POCT). Most available field isothermal amplifiers have been developed for single or two fluorescence channels [38]. However, Bio Molecular Systems (BMS, Australia) recently developed a 2 kg portable magnetic induction cycler (Mic) PCR machine with four real-time channels available. Thus, along with the Mic PCR Machine, the multiplex malaria Pan/Pf/Pv/IC LAMP assay, developed in the present study, can be used for POCT for diagnosing malaria species.

In the present study, we developed a multiplex malaria Pan/Pf/Pv/IC LAMP assay, which can classify malaria Pan, *P. falciparum*, and *P. vivax* within 40 min. On evaluating 118 malaria-infected clinical samples, the multiplex malaria Pan/Pf/Pv/IC LAMP assay revealed a performance similar to the commercial RealStar^®^ Malaria Screen & Type PCR Kit 1.0, which is based on the qPCR method. Thus, with portable multi-channel isothermal equipment, such as the Mic PCR Machine, the multiplex malaria Pan/Pf/Pv/IC LAMP assay can be applied in resource-limited settings for point-of-care diagnostics.

## Figures and Tables

**Table 1:** Primer sets used in the multiplex malaria Pan/Pf/Pv/IC LAMP.

Target	Name	Sequence (5′-3′)	Length (mer)	Reference
Malaria pan (18S rRNA gene)	M. pan F3	GTA TCA ATC GAG TTT CTG ACC	21	[31]
	M. pan B3	CTT GTC ACT ACC TCT CTT CT	20	
	M. pan FIP	TCG AAC TCT AAT TCC CCG TTA CCT ATC AGC TTT TGA TGT TAG GGT	45	
	M. pan BIP	CGG AGA GGG AGC CTG AGA AAT AGA ATT GGG TAA TTT ACG CG	41	
	M. pan FLP	CGT CAT AGC CAT GTT AGG CC	20	
	M. pan BLP	AGC TAC CAC ATC TAA GGA AGG CAG	24	
	M. pan BLP probe	[FAM]-CGG GCC CGT ACA AAG GGA ACA CCC ACA CTC CGA GCT ACC ACA TCT AAG GAA GGC AG	56	
*Plasmodium falciparum* (LDH gene)	P. f F3	AAT TGT AAA CTT ACA TGC ATC AC	23	Present study
	P. f B3	TCA AAT TTA GCT TTT TCC TCA CT	23	
	P. f FIP	GCC CTT CTA ACA AGG TTG AGC AGC TGC TGC TAT TAT CGA AAT	42	
	P. f BIP	TAT GGA CAC TCC GAT ATA TTC GGT GTA ATT CGA TAA CTT GTT CAA CAC	48	
	P. f FLP	CAA ATC TTT AAG TAG GAT TCA GCC	24	
	P. f BLP	GGT ACA CCT GTT GTT TTA GGT GCT	24	
	P. f BLP probe	[HEX]-CGG GCC CGT ACA AAG GGA ACA CCC ACA CTC CGG GTA CAC CTG TTG TTT TAG GTG CT	56	
*Plasmodium* *vivax* (16S rRNA gene)	P. v F3	GGA ATG ATG GGA ATT TAA AAC CT	23	[30]
	P. v B3	ACG AAG TAT CAG TTA TGT GGA T	22	
	P. v FIP	CTA TTG GAG CTG GAA TTA CCG CTC CCA AAA CTC AAT TGG AGG	42	
	P. v BIP	AAT TGT TGC AGT TAA AAC GCT CGT AAG CTA GAA GCG TTG CT	41	
	P. v FLP	GCT GCT GGC ACC AGA CTT	18	
	P. v BLP	AGT TGA ATT TCA AAG AAT CG	20	
	P. v BLP probe	[Texas red]-CGG GCC CGT ACA AAG GGA ACA CCC ACA CTC CGA GTT GAA TTT CAA AGA ATC G	52	
Human (actin gene)	IC F3	AGT ACC CCA TCG AGC ACG	18	[32]
	IC B3	AGC CTG GAT AGC AAC GTA CA	20	
	IC FIP	GAG CCA CAC GCA GCT CAT TGT ATC ACC AAC TGG GAC GAC A	40	
	IC BIP	CTG AAC CCC AAG GCC AAC CGG CTG GGG TGT TGA AGG TC	38	
	IC FLP	TGT GGT GCC AGA TTT TCT CCA	21	
	IC BLP	CGA GAA GAT GAC CCA GAT CAT GT	23	
	IC BLP probe	[Cy5]-CGG GCC CGT ACA AAG GGA ACA CCC ACA CTC CGC GAG AAG ATG ACC CAG ATC ATG T	55	
Quencher probe 1 Quencher probe 2		GAG TGT GGG TGT TCC CTT TGT ACG GGC CCG -BHQ1	30	
		CCT ACC CTC GTC CTA ACA CGG GAG CCT GCA CTG AC -BHQ2	35	

**Table 2:** Limit of detection (LOD) tests of monoplex and multiplex malaria Pan/Pf/Pv/IC LAMP assay for malaria Pan, *Plasmodium falciparum*, *Plasmodium vivax*, and internal control vectors (range: 10^7^–10^1^).

			Ct Values						
			Vector Dilution (Copies/µL)						
	**Primer set**	**Vector**	**10^7^**	**10^6^**	**10^5^**	**10^4^**	**10^3^**	**10^2^**	**10^1^**
**Monoplex** **LAMP**	**Pan (FAM)**	**Malaria Pan**	8.08	9.10	10.21	11.04	12.82	16.26	Neg
	**Pf (HEX)**	***P. falciparum***	10.44	11.21	12.59	13.30	17.73	21.26	Neg
	**Pv (Tex)**	***P. vivax***	12.05	14.22	15.48	16.00	19.77	22.60	Neg
	**IC (Cy5)**	**IC**	14.26	16.18	18.00	19.62	24.28	Neg	Neg
**Multiplex** **LAMP**	**Pan (FAM)** **+ Pf (HEX)** **+ Pv (Tex)** **+ IC (Cy5)**	**Malaria Pan**	12.15	13.78	15.55	17.28	19.10	22.81	Neg
		***P. falciparum***	17.79	19.00	20.53	26.65	31.48	31.67	Neg
		***P. vivax***	13.26	15.65	17.03	17.93	25.73	28.58	Neg
		**IC**	15.58	18.01	20.14	22.33	23.91	Neg	Neg

**Table 3:** Limit of detection (LOD) tests for monoplex and multiplex malaria Pan/Pf/Pv/IC LAMP assays and RealStar^®^ Malaria Screen & Type PCR Kit 1.0 using clinical malaria whole blood samples (range: 10^−1^–10^−6^).

Clinical Samples			Whole Blood Clinical Sample											
		Dilution *	10^−1^		10^−2^		10^−3^		10^−4^		10^−5^		10^−6^	
			CT	RFU	CT	RFU	CT	RFU	CT	RFU	CT	RFU	CT	RFU
*P. falciparum*	Monoplex LAMP	Pan (FAM)	9.86	15,145	10.83	16,032	12.68	17,075	14.43	17,203	Neg	-	Neg	-
		Pf (HEX)	9.71	16,795	10.82	16,581	13.73	17,218	Neg	-	Neg	-	Neg	-
	Multiplex LAMP	Pan (FAM)	16.70	7543	18.24	7629	20.70	8057	30.85	8118	Neg	-	Neg	-
		Pf (HEX)	21.16	3928	22.88	3969	24.72	4108	38.28	4020	Neg	-	Neg	-
		Pv (Tex)	Neg	-	Neg	-	Neg	-	Neg	-	Neg	-	Neg	-
		IC (Cy5)	24.71	4603	37.16	2616	Neg	-	Neg	-	Neg	-	Neg	-
	RealStar^®^ Malaria S&T PCR Kit 1.0	Pf (FAM)	22.61	11,985	27.09	12,807	30.82	12,548	35.41	11,329	39.26	7558	Neg	-
		Pv (Cy5)	Neg	-	Neg	-	Neg	-	Neg	-	Neg	-	Neg	-
*P. vivax*	Monoplex LAMP	Pan (FAM)	16.12	13,226	18.05	14125	32.51	13,617	37.25	12,230	Neg	-	Neg	-
		Pv (Tex)	12.80	9561	14.59	9454	Neg	-	Neg	-	Neg	-	Neg	-
	Multiplex LAMP	Pan (FAM)	19.36	7347	20.90	7527	25.27	7546	Neg	-	Neg	-	Neg	-
		Pf (HEX)	Neg	-	Neg	-	Neg	-	Neg	-	Neg	-	Neg	-
		Pv (Tex)	18.10	5398	20.08	5360	21.99	5541	Neg	-	Neg	-	Neg	-
		IC (Cy5)	54.86	1138	55.03	1124	Neg	-	Neg	-	Neg	-	Neg	-
	RealStar^®^ Malaria S&T PCR Kit 1.0	Pf (FAM)	Neg	-	Neg	-	Neg	-	Neg	-	Neg	-	Neg	-
		Pv (Cy5)	29.37	5712	33.71	5585	36.90	5049	Neg	-	Neg	-	Neg	-
*P. malariae*	Monoplex LAMP	Pan (FAM)	11.11	15,757	12.61	17,887	14.87	16,582	15.80	17,883	Neg	-	Neg	-
	Multiplex LAMP	Pan (FAM)	20.05	7706	22.29	7741	26.23	7531	Neg	-	Neg	-	Neg	-
		Pf (HEX)	Neg	-	Neg	-	Neg	-	Neg	-	Neg	-	Neg	-
		Pv (Tex)	Neg	-	Neg	-	Neg	-	Neg	-	Neg	-	Neg	-
		IC (Cy5)	32.60	6815	37.41	6486	38.92	6449	Neg	-	Neg	-	Neg	-
	RealStar^®^ Malaria S&T PCR Kit 1.0	Pm (FAM)	24.56	13,326	28.16	12,145	31.22	1875	34.67	11,985	36.91	5500	Neg	-
		Po (Cy5)	Neg	-	Neg	-	Neg	-	Neg	-	Neg	-	Neg	-
		Pk (Tex)	Neg	-	Neg	-	Neg	-	Neg	-	Neg	-	Neg	-
*P. ovale*	Monoplex LAMP	Pan (FAM)	16.60	15,880	Neg	-	Neg	-	Neg	-	Neg	-	Neg	-
	Multiplex LAMP	Pan (FAM)	22.20	7885	Neg	-	Neg	-	Neg	-	Neg	-	Neg	-
		Pf (HEX)	Neg	-	Neg	-	Neg	-	Neg	-	Neg	-	Neg	-
		Pv (Tex)	Neg	-	Neg	-	Neg	-	Neg	-	Neg	-	Neg	-
		IC (Cy5)	26.27	6097	Neg	-	Neg	-	Neg	-	Neg	-	Neg	-
	RealStar^®^ Malaria S&T PCR Kit 1.0	Pm (FAM)	Neg	-	Neg	-	Neg	-	Neg	-	Neg	-	Neg	-
		Po (Cy5)	34.12	2516	Neg	-	Neg	-	Neg	-	Neg	-	Neg	-
		Pk (Tex)	Neg	-	Neg	-	Neg	-	Neg	-	Neg	-	Neg	-

* The test was performed with whole blood clinical DNA samples, which were serially diluted with nuclease-free water.

**Table 4:** Comparison of clinical performance between the multiplex malaria Pan/Pf/Pv/IC LAMP assay and RealStar^®^ Malaria Screen & Type PCR Kit 1.0 for *Plasmodium* species (*P. falciparum*, *P. vivax*, *P. malariae*, and *P. ovale*) using clinical samples.

Clinical Samples		Multiplex Malaria Pan/Pf/Pv/IC LAMP Assay				RealStar^®^ Malaria Screen & Type PCR Kit 1.0					
		Pan (FAM)	Pf (HEX)	Pv (Tex)	IC (Cy5)	Pf (FAM)	Pv (Cy5)	IC (HEX)	Pm (FAM)	Po (Cy5)	IC (HEX)
*P. falciparum* (n = 61)	P/N	61/0	61/0	0/61	46/15	61/0	1/60	58/3	-	-	-
	Sensitivity	100%	100%	-	75.41%	100%	-	95.08%	-	-	-
	Specificity	-	-	100%	-	-	98.36%	-	-	-	-
*P. vivax* (n = 54)	P/N	54/0	0/54	54/0	44/10	0/54	54/0	54/0	-	-	-
	Sensitivity	100%	-	100%	81.48%	-	100%	100%	-	-	-
	Specificity	-	100%	-	-	100%	-	-	-	-	-
*P. malariae* (n = 2)	P/N	2/0	0/2	0/2	2/0	-	-	-	2/0	0/2	2/0
	Sensitivity	100%	-	-	100%	-	-	-	100%	-	100%
	Specificity	-	100%	100%	-	-	-	-	-	100%	-
*P. ovale* (n = 1)	P/N	1/0	0/1	0/1	1/0	-	-	-	1/0	0/1	1/0
	Sensitivity	100%	-	-	100%	-	-	-	100%	-	100%
	Specificity	-	100%	100%	-	-	-	-	-	100%	-
Non-infection (n = 90)	P/N	0/90	0/90	0/90	90/0	0/90	0/90	90/0	-	-	-
	Sensitivity	-	-	-	100%	-	-	100%	-	-	-
	Specificity	100%	100%	100%	-	100%	100%	-	-	-	-

P/N: positive/negative ratio.

## Data Availability

Data is contained within the article.
